# A study on the characteristics of cognitive function in patients with multiple system atrophy in China

**DOI:** 10.1038/s41598-021-84393-5

**Published:** 2021-03-02

**Authors:** Nannan Li, Tianwen Yang, Weizheng Ran, Xinning Zhang, Yao Wang, Zhifang Xu, Shan Ren, Qianyu Zhao, Bingyu Guo, Sushi Wang, Fanxing Meng, Zhigang Chen

**Affiliations:** 1 Department of Neurology, Dongfang Hospital, Beijing University of Chinese Medicine, Fengtai District, Beijing, 100078 China; 2 Beijing University of Chinese Medicine, Beijing, China; 3 Department of Acupuncture and Moxibustion, First Medical Center, Chinese PLA General Hospital, Beijing, China; 4 Acupuncture Research Center, Tianjin University of Traditional Chinese Medicine, Tianjin, China; 5 Beijing Hospital of Traditional Chinese Medicine, Beijing, China; 6 Guang’anmen Hosipital China Academy of Chinese Medical Sciences, Beijing, China

**Keywords:** Cognitive neuroscience, Neuroscience, Diseases of the nervous system, Parkinson's disease

## Abstract

Nonmotor symptoms in patients with multiple system atrophy (MSA) have received an increasing amount of attention in recent years, but no research on MSA patients' cognitive characteristics has been conducted in China. To evaluate the cognitive function of MSA patients in China. Using a case–control study design, 256 MSA patients and 64 controls were evaluated by the Montreal cognitive assessment (MoCA) scale to characterize their cognitive function. Like the controls, 60.5% of the patients with MSA had cognitive impairment, but the characteristics of cognitive impairment between the two groups were different. The cognitive impairment in MSA patients was prominent in the cognitive domains of visuospatial/executive functions, naming, attention, and orientation; particularly, the visuospatial/executive functions were the most significantly impaired, while impairment in language function was mainly seen in the controls. Besides, impairments in visuospatial/executive functions, attention, language, and orientation were more prominent in MSA-P (MSA with predominant Parkinsonism) patients than in MSA-C (MSA with predominant cerebellar ataxia). The cognitive impairments were more severe in patients with probable MSA than in patients with possible MSA. In addition, the results showed that the level of cognitive function was negatively correlated with the severity of MSA. This study, which characterized the cognitive function of MSA patients with the largest sample size known so far in China, found that patients with MSA do have cognitive impairment and display specific characteristics. Therefore, the cognitive impairment of MSA should be paid more attention.

The study has been registered in the Chinese Clinical Trial Registry (ChiCTR) (Registration No: ChiCTR1900022462).

## Introduction

Multiple system atrophy (MSA), a delayed sporadic progressive neurodegenerative disease, is a type of atypical Parkinsonian syndrome characterized by autonomic nervous system dysfunction, Parkinson-like symptoms that respond poorly to chronic levodopa therapy, and cerebellar ataxia and pyramidal symptoms.

The motor symptoms are remarkable in MSA and have received a significant amount of attention. Nevertheless, in recent years, nonmotor symptoms such as urinary dysfunction and sleep disorders associated with MSA, which seriously reduce the patients' quality of life, have begun to receive more attention^1,2^. Still, cognitive impairment in patients with MSA has a significant impact on the patients and their caregivers and is not well understood. Besides, cognitive impairment was considered a warning sign for MSA's diagnosis in both the 1999 and 2008 consensuses on MSA. As research has advanced in recent years, an increasing amount of evidence has shown that the cognitive impairment of patients with MSA cannot be ignored. More and more studies have shown that MSA patients have different degrees of cognitive impairment^3–7^. A study conducted by the neuropsychological working group of the MDS-MSA research group found that MSA patients' cognitive impairments were most prominent in executive functions and verbal fluency, followed by attention, memory, and visuospatial functioning^8^. The severity of cognitive impairment in MSA patients was similar to that caused by Parkinson’s disease and less severe than progressive supranuclear palsy (PSP)^9,10^. The rate of cognitive impairment gradually increases with the disease course's length, and the impairment gradually deteriorates with disease progression^11^. Moreover, cognitive impairment occurs in the two clinical MSA types [i.e., MSA-P (MSA with predominant Parkinsonism) and MSA-C (MSA with predominant cerebellar ataxia)] but manifests differently in the two types of patients. Attention deficit is predominant only in patients with MSA-C^12^. Compared with MSA-C, patients with MSA-P have more severe and extensive cognitive impairments^13^.

The abovementioned studies were mostly conducted in Europe, the United States, Japan, and South Korea. Currently, there is no large sample study on the cognitive function of patients with MSA in China to arouse experts' attention in related fields on the cognitive impairment with MSA. Therefore, this study used the largest sample size known to characterize the cognitive function in Chinese MSA patients. In addition, this study recruited age-, sex-, and education-matched controls for comparative analysis to yield more objective and accurate findings. This study emphasizes that the cognitive impairment of patients with MSA should be taken seriously.

## Methods

### Subjects

All 320 study participants [256 MSA patients and 64 controls (4:1 ratio)] visited the Dongfang Hospital of Beijing University of Chinese Medicine between September 2014 and March 2019. The two groups were matched based on age, sex, and education level.

All included MSA patients were diagnosed with possible or probable MSA according to the consensus diagnostic criteria^14^ and were between 30 and 80 years of age. The exclusion criteria were (1) a similar family history, (2) MSA caused by systemic disease or other confirmed causes, (3) hallucinations unrelated to drugs, (4) neurologically confirmed dementia according to the DSM-IV diagnostic criteria, (5) remarkably slow vertical saccades or vertical supranuclear gaze palsy, (6) local cerebral cortical lesions such as aphasia, body integrity identity disorder (BIID), and parietal dysfunction, or (7) pregnant patients or patients who could not complete the scale test and related examinations due to severe heart, liver, or kidney diseases.

The controls were between 30 and 80 years of age. The exclusion criteria were (1) central nervous system diseases that could lead to progressive memory and cognitive deficiencies, including Alzheimer’s disease, vascular dementia, Parkinson’s disease, Huntington’s disease, subdural hematoma, normal-pressure hydrocephalus, and brain tumor, (2) systemic diseases known to cause dementia, including hypothyroidism, folic acid or vitamin B12 deficiency, niacin deficiency, neurosyphilis, and HIV infection, (3) a history of severe craniocerebral trauma with persistent neurological defects or confirmed brain structural abnormalities, (4) a history of alcohol and drug abuse; or (5) pregnant patients or patients who could not complete the scale test and related examinations due to severe heart, liver, or kidney diseases.

### Assessment

All subjects received a series of clinical examinations and professional assessments. The demographic data (age, sex, and education level) of all study subjects and the clinical characteristics (age of onset, disease duration of MSA, Unified Multiple System Atrophy Rating Scale (UMSARS) I, II, IV scores, and Montreal cognitive assessment (MoCA) scale scores, all determined by professional neurologists) were collected.

The original MoCA scores were converted into MMSE scores^15^. The total MoCA scores were divided into 0 to 3 for severe cognitive impairment, 4 to 13 for moderate cognitive impairment, and 14 to 21 for mild cognitive impairment. The total MoCA scores between 22 and 30, which indicate normal cognitive functioning according to the conversion criteria, were further divided into two categories (22 to 25 and 26 to 30) for more detailed analysis because the MoCA scale has a cutoff of 26 for cognitive impairment.

### Ethics

This study was reviewed and approved by the Ethics Committee of Dongfang Hospital of Beijing University of Chinese Medicine (No. JDF-IRB-2018035602). All research was performed in accordance with the relevant guidelines and regulations. Written informed consent was obtained for each participant according to the institutional guidelines.

### Data analysis

SPSS 25.0 was used for statistical analysis in this study. Continuous data are mostly expressed as means ± standard deviations, with some data expressed as medians and interquartile ranges. The Mann–Whitney *U* test was used to compare the data that were not normally distributed between the two groups. The Spearman rank correlation analysis was used to analyze the correlation between MoCA scores and the patients' clinical characteristics. *P* < 0.05 indicated that the difference was statistically significant.

## Results

### Demographic characteristics

All 320 subjects (256 MSA patients and 64 controls) received clinical neurological examinations and were evaluated using the MoCA scale. The basic demographic data and clinical characteristics of the study participants are presented in Table 1. There were no differences in age (z = 1.26, *P* = 0.21) or education duration (z = − 0.45, *P* = 0.66) between the two groups. The number of females was smaller than that of males in both groups, and the difference in sex ratio was not significant between the two groups (χ^2^ = 0.01, *P* = 0.91).

**Table 1:** Demographic data and clinical characteristics of MSA patients and controls.

Clinical characteristic	MSA patients (n = 256)	Controls (n = 64)	P value
Age (years)	56.6 (7.89)	57.61 (4.7)	0.21
Education duration (years)	12.0 (3.91)	11.95 (3.1)	0.66
Sex (female) (%)	41.4	42.2	0.91
Total MoCA score	23.60 (4.6)	24.27 (3.0)	0.80
Duration of disease (years)	3.34 (1.8)	–	
UMSARS I	21.23 (6.5)	–	
UMSARS II	19.92 (8.9)	–	
UMSARS IV^a^	2.0 (2)	–	
UMSARS total score (I+II)	41.15 (14.84)	–	

Data are expressed as mean (standard deviation). ^a^The data are expressed as median (interquartile range).

### There were no significant differences in MoCA scores between MSA patients and controls

The clinical neurological examinations revealed that 60.5% of the MSA patients and 59.4% of the controls had cognitive impairment. The total MoCA scores' differences were not significant between the two groups (z = 0.26, P = 0.80). The sex ratio difference among participants with cognitive impairment was not significant between the two groups (χ^2^ = 0.03, P = 0.86).

### The cognitive impairment characteristics of MSA patients were different from those of controls

Table 2 shows the mean MoCA scores of MSA patients and controls. Even though there were no significant differences in the total MoCA scores, the two groups had cognitive impairments in different cognitive domains (z = 0.26, *P* = 0.80). The MSA patients mostly had impaired visuospatial/executive functions (z = 3.76, *P* < 0.01), naming (z = 3.03, *P* < 0.05), attention (z = 3.49, *P* < 0.01), and orientation (z = 2.83, *P* < 0.05), while the cognitive decline of controls was mostly in the language (z = -2.24, *P* < 0.05) and abstraction (z = -3.72, *P* < 0.01) domains.

**Table 2:** MoCA scores of MSA patients and controls.

Item	MSA patients (n = 256)	Controls (n = 64)	P
Total MoCA score (max = 30)	23.6 (4.6)	24.3 (3.0)	0.80
Visuospatial/executive (max = 6)	3.3 (1.5)	4.1 (1.1)	< 0.01
Naming (max = 3)	2.8 (0.5)	3.0 (0.2)	< 0.01
Attention (max = 6)	4.9 (1.3)	5.4 (0.9)	< 0.01
Language (max = 3)	2.0 (0.9)	1.8 (0.7)	0.03
Abstraction (max = 2)	1.5 (0.7)	1.1 (0.8)	< 0.01
Delayed recall (max = 5)	3.1 (1.6)	2.7 (1.6)	0.13
Orientation (max = 6)	5.5 (0.9)	5.8 (0.5)	0.01

Data are expressed as mean (standard deviation), and “max” indicates the highest score for the item.

**Figure 1:**
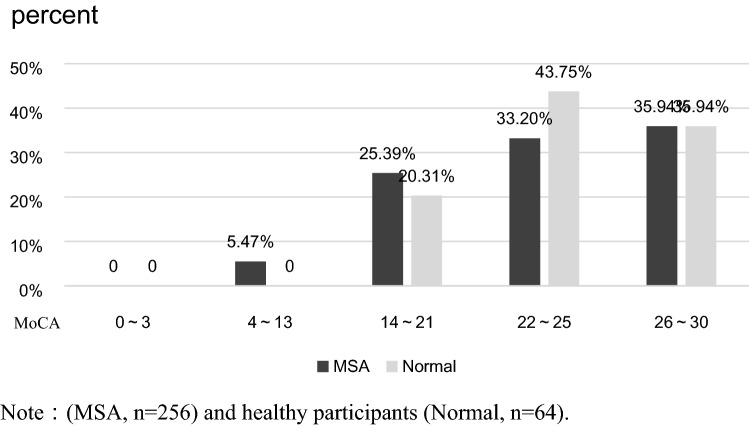
Total MoCA score distribution for MSA patients. Note: (MSA, n = 256) and controls (normal, n = 64).

The distribution of the total MoCA scores for MSA patients and controls is shown in Fig. 1. As shown in Fig. 1, 5.5% of the MSA patients had moderate cognitive impairment, while the controls' cognitive impairment was mostly mild. In addition, 33.2% of the MSA patients and 43.8% of the controls had a total MoCA score between 22 and 25. The two groups had a similar overall total MoCA score distribution (z = − 1.03, P = 0.30).

### Different types and diagnostic stratification of MSA patients have different characteristics of cognitive impairment

Table 3 shows the characteristics of cognitive impairment in patients with MSA with different symptoms and diagnostic categories. Compared with patients with MSA-C, those with MSA-P were older (*P* < 0.001) and showed a higher proportion of females (*P* = 0.001), but without a difference in education level (*P* = 0.137). The cognitive impairment in MSA-P patients was significantly more severe than that in MSA-C patients (z = − 2.84, *P* < 0.05). More specifically, MSA-P patients had more severe impairments in visuospatial/executive functions (z = − 2.00, *P* < 0.05), attention (z = − 2.35, *P* < 0.05), language (z = − 2.01, *P* < 0.05), and orientation (z = − 3.66, *P* < 0.01) than did MSA-C patients. Further, the cognitive function of patients with different diagnostic categories of MSA was compared. Compared to the patients with possible MSA, patients with probable MSA had more severe impairment in language function (z = − 2.121, *P* < 0.05) and had impairments of similar severity in other cognitive abilities (*P* > 0.05).

**Table 3:** Characteristics of cognitive impairment in patients with MSA in different symptom and diagnostic categories.

Item	Symptom categories			Diagnostic categories		
	MSA-C (n = 171)	MSA-P (n = 85)	P	Possible MSA (n = 67)	Probable MSA (n = 189)	P
Age of onset	52.8 (7.2)	56.2 (7.1)	< 0.001			
Sex (male/female)	113/58	37/48	0.001			
Education level	12 (9, 15)	12 (9, 15)	0.137			
Total MoCA score	24.2 (4.3)	22.4 (5.0)	< 0.01	24.2 (3.7)	23.4 (4.9)	0.39
Visuospatial/executive	3.4 (1.4)	3.0 (1.5)	< 0.05	3.4 (1.4)	3.3 (1.5)	0.57
Naming	2.8 (0.4)	2.8 (0.5)	0.43	2.8 (9.4)	2.8 (0.5)	0.59
Attention	5.0 (1.2)	4.6 (1.4)	0.02	5.0 (1.0)	4.8 (1.4)	0.66
Language	2.0 (0.8)	1.8 (1.0)	< 0.05	2.1 (0.9)	1.9 (0.9)	0.03
Abstraction	1.5 (0.7)	1.5 (0.8)	0.70	1.4 (0.8)	1.6 (0.7)	0.12
Delayed recall	3.2 (1.6)	2.9 (1.5)	0.13	3.2 (1.4)	3.0 (1.6)	0.85
Orientation	5.6 (0.9)	5.3 (0.9)	< 0.01	5.6 (0.7)	5.5 (0.9)	0.63

Data are expressed as mean (standard deviation).

Table 4 shows the clinical characteristics of MSA patients with or without cognitive impairment. Total MoCA scores < 26 and ≥ 26 indicate cognitive impairment and normal cognitive function, respectively. Compared to the MSA patients with normal cognitive function, MSA patients with cognitive impairment were older (z = − 2.99, *P* < 0.01), had shorter education duration (z = 3.81, *P* < 0.01), and had more severe MSA, i.e., higher UMSARS I, II, and IV scores (*P* < 0.01).

**Table 4:** Clinical characteristics of MSA patients with or without cognitive impairment.

Clinical characteristics	MSA patients with cognitive impairment (n = 155)	MSA patients with normal cognitive function (n = 101)	P value
Age (years)	57.8 (7.3)	54.9 (8.5)	< 0.01
Education duration (years)	11.1 (4.2)	13.3 (2.9)	0.32
Sex (female) (%)	41.9	40.6	< 0.01
Duration of disease (years)	3.5 (1.9)	3.2 (1.7)	< 0.01
UMSARS I	22.6 (6.7)	19.0 (5.4)	< 0.01
UMSARS II	22.2 (8.9)	16.3 (8.9)	< 0.01
UMSARS IV^a^	3.0 (2.0)	2.0 (2.0)	< 0.01

Data are expressed as mean (standard deviation).

^a^The data are expressed as median (interquartile range).

### Analysis of influencing factors of cognitive impairment in MSA patients

Correlations between total MoCA score and clinical characteristics of MSA patients were assessed using Spearman rank correlation analysis. Factors such as age, disease duration, education duration, sex (female/male), and disease severity (UMSARS I, II, and IV scores) might affect the total MoCA score and thus were included in the analysis. The results showed no significant linear correlations between total MoCA score and sex and disease duration (*P* > 0.05).

**Figure 2:**
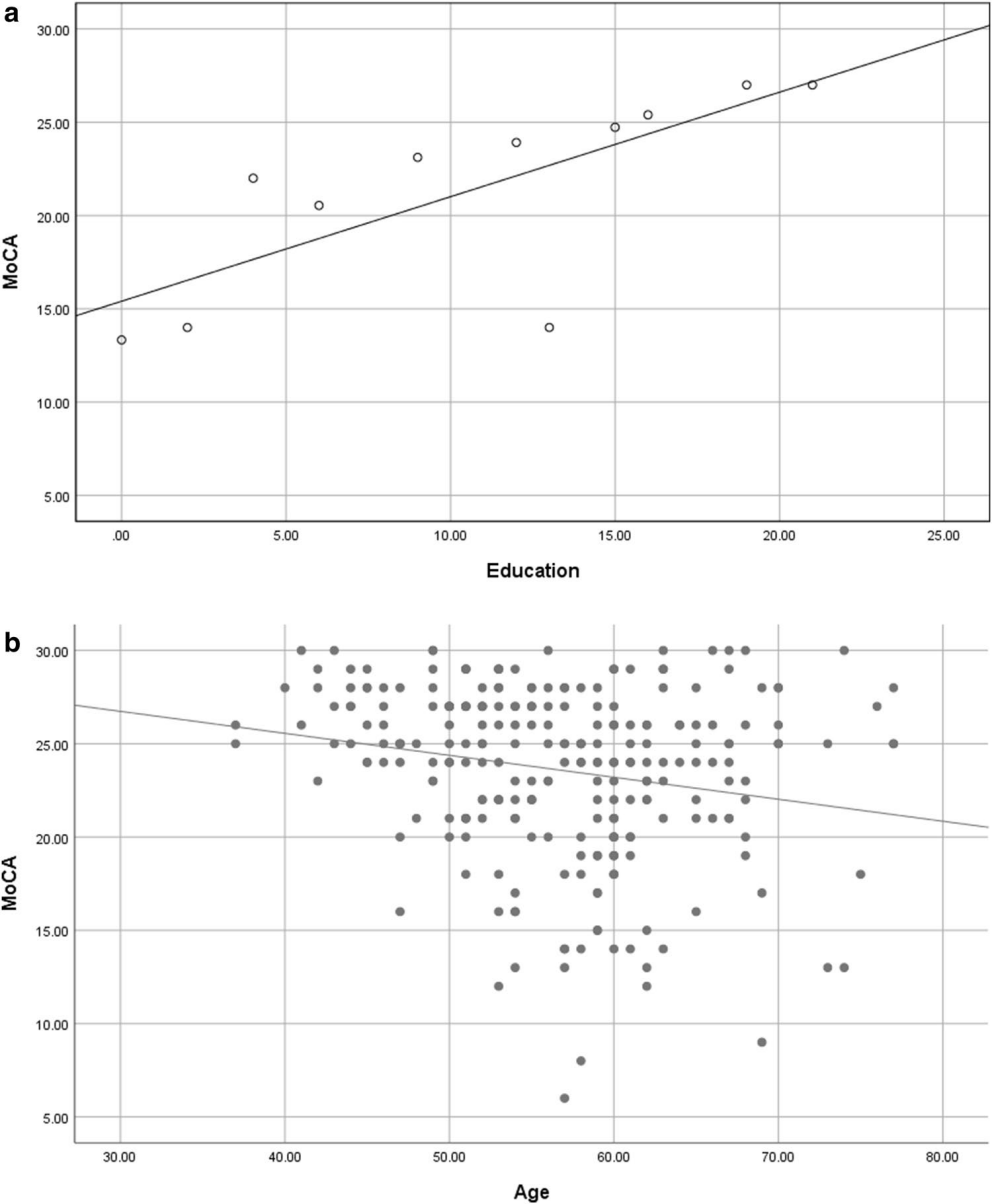
Correlations of total MoCA score with age and education duration for MSA patients (**A**,**B**). (**A**) Total MoCA score was positively correlated with education duration in MSA patients (r = 0.44, *P* < 0.01); (**B**) total MoCA score was negatively correlated with age in MSA patients (r = − 0.20, *P* < 0.01).

Figure 2 shows the correlations of the total MoCA scores with age and education duration for MSA patients. The total MoCA scores were positively correlated with education duration in MSA patients (r = 0.44, *P* < 0.01) (Fig. 2A). The total MoCA scores were negatively correlated with age in MSA patients (r = − 0.20, *P* < 0.01) (Fig. 2B).

**Figure 3:**
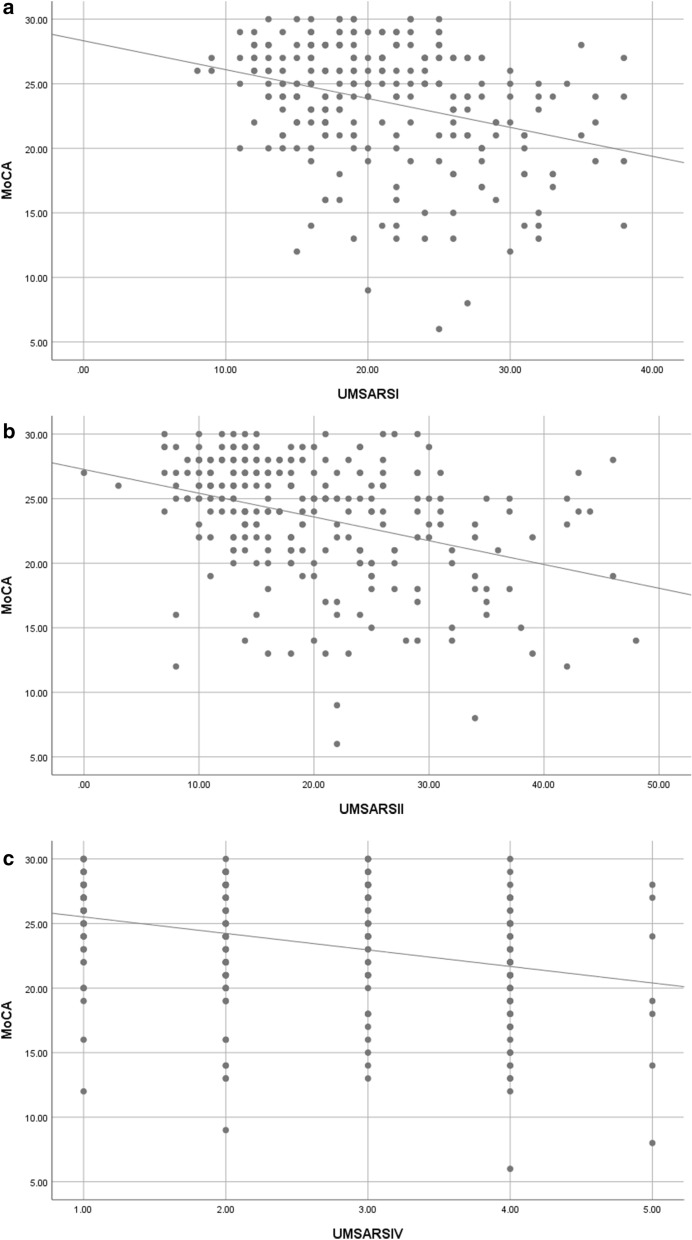
Correlations of total MoCA score with UMSARS I, II, and IV scores in MSA patients. (**A**) UMSARS I score (r = − 0.31, *P* < 0.01), (**B**) UMSARS II score (r = − 0.36, *P* < 0.01), (**C**) UMSARS IV score (r = **− **0.32, *P* < 0.01) were negatively correlated with total MoCA score, which suggests a possible correlation between the severity of the disease and degree of cognitive impairment.

Figure 3 shows the correlations of total MoCA scores with UMSARS I, II, and IV scores in MSA patients. UMSARS I scores (r = − 0.31, *P* < 0.01), UMSARS II scores (r = − 0.36, *P* < 0.01), and UMSARS IV scores (r = − 0.32, *P* < 0.01) were negatively correlated with the total MoCA scores, suggesting a possible correlation between the severity of the disease and the degree of cognitive impairment.

Table 5 shows that in MSA-C, age of onset (r = − 0.24, *P* = 0.002) and education level (r = 0.304, *P* < 0.001) were correlated with MoCA scores. In MSA-P, age of onset was not correlated with MoCA scores (r = 0.006, *P* = 0.960), while education level was (r = 0.341, *P* = 0.001).

**Table 5:** Spearman correlation between age of onset and education level and MoCA.

	r	P
**MSA-C**		
Age of onset	− 0.240	0.002
Education level	0.304	< 0.001
**MSA-P**		
Age of onset	0.006	0.960
Education level	0.341	0.001

## Discussion

This study investigated MSA patients' cognitive function status in China using a large sample, and all study subjects were managed strictly in accordance with the established practice standards. The results revealed that MSA patients had different degrees of cognitive impairment but no significant differences in the cognitive impairment rate between MSA patients and controls (60.5% and 59.4%, respectively). The prevalence of cognitive impairment in this study's controls differs from that reported by other studies in China that also used the MoCA scale as a cognitive assessment tool. For example, a large sample study with 7445 veterans over 60 years old in China found that 44.3% of the participants had cognitive impairment^16^. In another study involving 1027 participants with an average age of 72.5 years in Shanghai, China, the incidence of cognitive impairment was 30.9%^17^. The differences in results between the present study and previous studies might be because the present study's controls were geographically concentrated, and the sample size was insufficient. Besides, the controls were visiting the hospitals for various reasons that could affect the cognitive functions. Indeed, 35% of the controls had diabetes, which is associated with some level of cognitive dysfunction^18,19^.

Although the frequency of cognitive impairment was similar between MSA patients and controls, the ratio of severe cognitive impairment in the MSA group was higher than in the control group. In comparison, the ratio of mild cognitive impairment was lower. At the same time, there were significant differences in the areas of cognitive impairment between the two groups. The cognitive impairments of the controls were mainly in the language and abstraction domains. In contrast, MSA patients had cognitive impairments mostly in visuospatial/executive functions, naming, attention, and orientation, primarily visuospatial/executive functions, which confirmed previous research findings on MSA-associated cognitive changes^5,8,13^.

The changes in the cognitive domains involved in MSA-associated cognitive impairment can be explained by imaging technologies. Cognitive impairment in MSA patients is triggered by focal fronto-striatal degeneration and is characterized by abnormalities in the cortical and subcortical structures^20,21^. MSA patients have extensive cortical (bilateral frontal lobe, occipito-temporal area, and parietal area), subcortical and white matter changes, and MSA patients with cognitive impairment often show a focal volume reduction in the left dorsolateral prefrontal cortex^22^. A similar study also found that patients with MSA-C have cortical changes in the frontal, temporal, and parietal areas and atrophy of the bilateral thalamus, left cerebellum, and left hippocampus and that these changes were significantly correlated with attention, execution, and visuospatial dysfunctions^23^. MSA-P patients have significant bilateral gray matter atrophy of the basal ganglia, cerebellum, and temporal and frontal cortical areas. The severity of their cognitive impairment is correlated with the volume of the neocortex, cerebellum, and striatum^24^.

Previous studies have reported that 20–33% of MSA patients have cognitive impairment^5,9,25^. In the present study, 60.5% of MSA patients had cognitive impairment, which was significantly higher than that reported by previous studies. A possible reason for this discrepancy is that previous studies mostly used the MMSE, FAB, and DRS scales and rarely used the highly sensitive MoCA scale. The MoCA scale is a sensitive tool for detecting cognitive impairment, especially in the elderly^16,17^, but it cannot be used to rate the severity of cognitive impairment. The MMSE scale is an effective tool to rate cognitive impairment, but it cannot detect mild cognitive impairment^16,17^. Therefore, the National Alzheimer’s Coordinating Center (NACC) research group of the United States of America proposed converting the MoCA scores into MMSE scores^15^. According to the NACC conversion method, we set the MoCA scores' cutoffs as follows: 0–3 for severe dementia, 4–13 for moderate dementia, and 14–21 for mild dementia. MoCA scores of 22–30, which are considered normal, were further divided into two categories (22–25 and 26–30) for a more detailed analysis, with a MoCA score of 26 as the cutoff for cognitive impairment because the MoCA scale has a higher sensitivity.

The present study revealed that the cognitive impairment in MSA patients was mainly characterized by mild cognitive deficiency, as evidenced by the fact that 33.2% of the MSA patients had a total MoCA score of 22 to 25. Patients with mild cognitive impairment are often overlooked when using the MMSE alone. Although we cannot rule out the possibility of ethnic differences, the high incidence rate of cognitive impairment in this study can be attributed to the MoCA use. The MoCA scale can accurately and comprehensively detect characteristics of cognitive functioning, especially for mild cognitive impairment; therefore, we highly recommend it for the clinical screening of cognitive impairment.

Furthermore, we compared the cognitive functions of patients with different clinical types (MSA-C and MSA-P) and different diagnostic categories of MSA. The results showed that both MSA-P and MSA-C patients had extensive cognitive impairment and that MSA-P patients had more deteriorated cognitive functions. Compared with MSA-C patients, MSA-P patients had more prominent impairment in various cognitive domains, including visuospatial/executive functions, attention, language, and orientation. Our findings were consistent with previous research results^13^. Notably, although verbal fluency may be lower in MSA patients than in PD patients^26^, the language impairment was not prominent in MSA patients than the controls, as discussed earlier. This study also found that language impairment was significantly more severe in MSA-P patients than in MSA-C patients. Nevertheless, the MoCA evaluates only repetition and verbal fluency and does not cover other aspects of language function involving natural phonetics, grammar, and lexical semantics. Therefore, further research is required to clarify whether language impairment is a significant manifestation of MSA-associated cognitive function decline.

MSA is divided into two diagnostic categories (possible and probable MSA) according to the impairment severity of autonomic nervous functions. This study revealed that the cognitive impairment in patients with probable MSA was significantly more significant than that in patients with possible MSA. Previous studies have found that cognitive function in MSA patients is independently associated with cardiovascular and neurological abnormalities^9^. In a study assessing the frontal lobe dysfunction in MSA patients using event-related brain potentials (ERPs), the patients with orthostatic hypotension (OH) scored lower in executive function and had a delayed novelty P3 somatosensory response when compared with those without OH, suggesting that OH is associated with frontal lobe dysfunction^27^. Results from the literature have confirmed the correlation between OH and cognitive impairment in patients with the α-synuclein disease (e.g., Parkinson’s disease, dementia with Lewy bodies, and MSA) and showed that both OH and cognitive impairment are caused by diffuse brain damage and α-synuclein deposition. OH-mediated cerebral hypoperfusion impairs cognitive function, and then the synergistic effect of the two leads to further cognitive impairment^28^. This might explain why cognitive impairment was more severe in patients with probable MSA in this study.

The large sample size of this study allowed us to identify the influencing factors of cognitive impairment in MSA patients in China. Age and education level are factors known to affect cognitive status. A study reported that male MSA patients are more prone to cognitive impairment and that patients with cognitive impairment often have suffered MSA for a longer time despite the weak correlation between disease duration and cognitive impairment^9^. On the other hand, the present study did not observe similar results. Cognitive impairment of MSA patients did not show significant sex differences and had no significant correlation with disease duration, and cognitive impairment even occurred at the early stage of MSA in some patients. MSA severity (UMSARS I, II, and IV scores) was negatively correlated with the cognitive function level, i.e., the patients with more severe MSA had more prominent cognitive impairment. This finding is consistent with previous research results^20,25^.

This study has some limitations. All patients and controls were from a single hospital, and there is the possibility of selection, practice, and geographical biases. In addition, there is a selection bias since the controls visited the hospital for various reasons and were not from the general population. Even if individuals with neuropsychiatric disorders and severe chronic conditions were excluded, it is nonetheless possible that those controls had worse cognitive functions than the general population. Second, the impact of medication, depression, anxiety, and sleep disorders were not taken into account. Indeed, some MSA patients have anxiety, depression, and sleep disorders based on our clinical experience. A search of the relevant literature showed that anxiety and depression have a high incidence in patients with MSA, but they are generally mild to moderate in severity^29–31^, and are, therefore, unlikely to have a substantial impact on cognitive functions^32,33^. Third, the use of MoCA alone for evaluation may indeed have certain disadvantages and biases. Based on this study and the literature^5,9,25^, we observed that MSA patients' cognitive impairment was mainly mild to moderate in severity. Therefore, we used the MoCA since the MoCA is sensitive to mild and moderate cognitive impairment^15^, while MMSE, ADAS-COG, and are not^16,17^. Fourth, the diagnosis of MSA was made clinically and based on imaging, but some patients might indeed have had other underlying diseases, but a autopsy was not performed in the present study, and all included patients had no overt diagnoses of Parkinson’s disease, dementia with Lewy body, or PSP. Finally, an exclusion criterion was a diagnosis of dementia, but some patients had MoCA scores ≤ 13 when we tested them in the present study. Nevertheless, they had no history of a diagnosis of dementia before participation. Therefore, we kept those patients in the analyses, but it might introduce some bias.

In conclusion, this study with a large sample size revealed that a substantial proportion of MSA patients in China suffered from significant cognitive impairment and different characteristics from controls’ cognitive impairment. The patients with different types of and different diagnostic categories of MSA suffered from different extents of cognitive impairment, and the severity of the condition might be an important contributor impacting on cognitive impairment. These results strongly suggest that cognitive impairment in MSA patients is characteristic, and cognitive impairment should be taken seriously in MSA's diagnosis. Especially, it should not be considered as an exclusion criterion or a warning sign. This study fills the gap in the research on the cognitive impairment characteristics of patients with MSA in China, and it provides relevant data on the cognitive characteristics of MSA patients in China, provides a reference for the study on the clinical characteristics of MSA, and it also indicated that the MoCA scale might be more appropriate for the study of cognitive impairment in MSA patients.

## Data Availability

All relevant data are presented in the paper
